# Neutrophil-to-lymphocyte and platelet-to-lymphocyte ratios in bacterial infections: contributions to diagnostic strategies in a tertiary care hospital in Tunisia

**DOI:** 10.12688/f1000research.146952.1

**Published:** 2024-08-29

**Authors:** Jihed Anoun, Mariem Ajmi, Salma Riahi, Yosra Dhaha, Donia Mbarki, Imen ben Hassine, Wiem Romdhane, Wafa Baya, Najah Adaily, Anis Mzabi, Fatma Ben Fredj, Amina Bouattay

**Affiliations:** 1 Faculty of Medicine, University of Sousse, Sousse, Tunisia; 2 Internal Medicine, Sahloul Hospital, Sousse, Sousse, 4011, Tunisia; 3 Laboratory of Hematology, Sahloul University Hospital, Sousse, Sousse, 4011, Tunisia; 4 Faculty of Pharmacy of Monastir, Monastir, Tunisia

**Keywords:** Infections, Neutrophil-to-lymphocyte ratio (NLR), Platelet-to-lymphocyte ratio (PLR), Diagnosis.

## Abstract

**Background:**

Bacterial infections continue to pose a global health challenge, driven by antibiotic resistance and septicemia. This study aimed to assess the diagnostic utility of neutrophil-to-lymphocyte ratio (NLR) and platelet-to-lymphocyte ratio (PLR) in bacterial infections versus non-infectious causes of inflammation.

**Methods:**

A prospective study included 164 adult patients who were divided into two groups: a group of patients with confirmed bacterial infections and a second group of patients with other diagnoses (inflammatory pathologies, neoplasms, venous thromboembolic diseases, etc.). NLR and PLR values were compared between the bacterial infection group and the non-infectious causes group and the diagnostic performances of NLR and PLR for detecting bacterial infections were evaluated in comparison with other infection markers.

**Results:**

NLR and PLR were significantly higher in bacterial infections (p < 10 ^^-6^), and NLR was correlated positively with inflammation markers. NLR and PLR demonstrated significant potential in diagnosing bacterial infections, with an AUC of 0.72 and 0.60, respectively, using the following cutoff values: 4.3 for NLR and 183 for PLR.

**Conclusion:**

These findings underscore the importance of NLR and PLR as adjunctive tools for bacterial infection diagnosis.

## Introduction

Bacterial infections are common conditions affecting millions of people worldwide. Due to the rise in antibiotic resistance and septicemia, bacterial infections remain one of the leading causes of death on a global scale. In 2019, deaths associated with bacterial infections accounted for 13.6% of all deaths. ^1^

Some infections are relatively benign, while others can be much more serious, requiring urgent medical intervention, even hospitalization. This presents a significant challenge for healthcare professionals.

The “gold standard” for diagnosing a bacterial infection involves isolating the pathogenic microorganism, but this approach is not always straightforward in routine practice. Despite the availability of numerous diagnostic tools, optimizing patient management remains fundamental for effective treatment. Several inflammatory markers have proven their effectiveness in diagnosing, prognosing, and assessing treatment response. These markers include C-reactive protein (CRP), white blood cell count, and procalcitonin (PCT). ^2^

Growing interest is emerging in new markers such as the neutrophil-to-lymphocyte ratio (NLR) and platelet-to-lymphocyte ratio (PLR). Their relevance stems from changes observed in the number of neutrophilic granulocytes, platelets, and lymphocytes during the inflammatory response. ^3^^,^^4^

In response to a bacterial infection, the number of neutrophilic granulocytes and platelets increases, while the number of lymphocytes decreases due to redistribution and increased apoptosis. ^4^ NLR and PLR prove to be a rapid, cost-effective, and applicable approach for early diagnosis, treatment, and prognosis of various diseases, such as cancer, community-acquired pneumonia, and sepsis. ^5^^–^^7^

The aim of this study was to investigate the diagnostic utility of NLR and PLR in bacterial infections compared to other non-infectious causes of inflammation.

## Methods

### Study population

To address the research question, a prospective comparative study was conducted within the internal medicine department. The study included a total of 164 patients admitted between January 1st and July 31st, 2023.

*Inclusion criteria*

• Adults aged 18 and older admitted to the internal medicine department between January 1st and July 31st, 2023.

*Exclusion criteria*

• Previous antibiotic treatment before admission (one week before admission).
• Patients taking immunosuppressive treatments.

### Laboratory values and clinical assessment

For each patient, the following parameters were recorded upon admission:

• Demographic data, medical history, and clinical symptoms.
• Results of laboratory analyses, including complete blood count (absolute neutrophil, platelet, and lymphocyte counts), and other inflammatory markers such as CRP and PCT (CRP with normal range < 5mg/L and PCT with normal range < 0.1 ng/ml); We considered PCT positive for infectious diseases when it was > 0.25 ng/ml).

NLR and PLR were calculated by dividing the number of neutrophils by the number of lymphocytes and the number of platelets by the number of lymphocytes, respectively. The normal range of neutrophil-to-lymphocyte ratio (NLR) and platelet-to-lymphocyte ratio (PLR) varies from 1 to 3 and 90 to 210, accordingly. ^8^ NLR and PLR values were compared between the bacterial infection group and the non-infectious causes group.

White blood cell (WBC) and differential cell counts were obtained using the DxH 900 hematology system by Beckman Coulter, California, USA.

### Statistical analysis

Statistical analysis was carried out using the software packages SPSS 21 (IBM SPSS Statistics for Windows, Version 21.0. Armonk, NY: IBM Corp) and MedCalc Statistical Software version 19.2.6 (MedCalc Software bv, Ostend, Belgium; https://www.medcalc.org; 2020) and appropriate statistical tests, such as the Mann-Whitney test and Kruskal-Wallis test, were applied as dictated by the data characteristics and study requirements.

The significance level for statistical analysis was set at p < 0.05, denoting that differences between groups and the strength of parameter correlations were assessed against this predetermined threshold.

The correlation coefficient (r) was employed to measure the strength and direction of linear relationships between parameters. An r-value close to 1 indicated a strong positive correlation, while an r-value close to -1 indicated a strong negative correlation. An r-value close to 0 suggested a weak or non-existent correlation between parameters.

The significance levels were indicated as follows:

a p < 0.05
b p < 0.01
c p < 0.001

ROC curves were plotted to compare the diagnostic accuracy of NLR, PLR, CRP, and PCT in the context of bacterial infections versus non-infectious causes. The diagnostic performances of NLR, CRP, and PCT for detecting bacterial infections were evaluated by calculating sensitivity and specificity.

### Ethical considerations

- The prior agreement of the departments’ chiefs concerned with the study (the internal medicine department and the hematology laboratory) was obtained, as well as the initial approval from the ethics committee of Sahloul University Hospital of Sousse on May 11, 2022, under the number **HS 22-2022**, this approval confirms that there are no ethical issues concerning the work.
- Written and verbal consent were obtained from all participants before their involvement in the study, ensuring that their rights, privacy, and confidentiality were rigorously upheld throughout the research process.

## Results

A total of 164 patients were included in the study. The study population was divided into two groups: a group of patients with confirmed bacterial infections and a second group of patients with other diagnoses (inflammatory pathologies, neoplasms, venous thromboembolic diseases, etc.). The median age within this study population was 50 years (ranging from 37 to 66 years), with a male-to-female ratio of 0.76. The epidemiological characteristics and the mean rates of NLR, PLR, PCT and CRP are described in Table 1.

**Table 1.** The epidemiological characteristics and the median rates of NLR, PLR, PCT and CRP of the Study Population by Disease Type.

Groups	Subgroups	Sample size (n)	Median age (years) (IQR)	Gender Ratio	Median NLR (IQR)	Median PLR (IQR	Median PCT (ng/ml) (IQR)	Median CRP (mg/l) (IQR)
*Infections (n=53)*	Bacterial	47	59 [38-71]	0.89	6.5[3-10.3]	204.3 [106.4-298.75]	0.33[0.07-1.68]	144 [26.5-187]
	Viral	4	-	-	-	-	-	-
	Parasitic	2	-	-	-	-	-	-
*Non-Infectious Diseases (n=111)*	Inflammatory Diseases	55	40 [30-56]	0.53	3.1 [1.9-4.8]	142.5 [99.35-241.24]	0.048 [0.03-0.1]	5 [2-55]
	Neoplasms	11	69 [51-76]	2.66	7.5 [3.77-12]	181.47[77.4-289.1]	0.9[0.1-1.2]	88 [36-188]
	Venous Thromboembolic Diseases	6	51[38.5-67.75]	2	3.2 [2.18-3.45]	121.3 [105.9-144.1]	0.04 [0.03-0.05]	7.5 [4.25-40.75]
	Other	39	51 [41-65]	0.75	2 [1.3-3.2]	122.73 [104.21-152]	0.05 [0.03-0.08]	2 [2-8.5]

NLR: neutrophil-to-lymphocyte ratio; PLR: platelet-to-lymphocyte ratio; CRP: C-reactive protein; PCT: procalcitonin.

Our findings revealed that bacterial infections are linked to a notable increase in the NLR to 9.7 (normal range from 1 to 3) and the PLR to 218.14 (normal range from 90 to 210). Additionally, patients with neoplasms also demonstrate elevated PLR and NLR.

Our results revealed significant variations in NLR based on gender. Indeed, men generally had slightly higher NLR values than women, with means of 7.2 in men and 4.7 in women (p<10 ^-6^). In addition to gender-based variations, we also observed significant differences in NLR based on age. Specifically, older subjects had higher NLR values, with a mean of 8.8 compared to 4.3 in younger individuals (p=0.001).

In the group of patients with bacterial infections, there were significantly higher levels of NLR, PLR, PCT, CRP, and absolute neutrophil count (ANC) compared to those with non-infectious diseases (p<10 ^-6^) (see Table 2).

**Table 2.** Comparison of median parameters (NLR, PLR, ANC, PCT, CRP) between bacterial infections and non-infectious diseases.

Parameter studied	Bacterial Infections (n=47)	Non-Infectious Diseases (n=111)	p-Value ^*^
*NLR*	6.5 [3-10.3]	3.1 [1.7-4.2]	p<10 ^-6c^
*PLR*	204.3 [106.4-298.75]	133.68 [106.21-225.22]	p=0.049 ^a^
*ANC (WBCs/μL)*	7500 [4450-12400]	4300 [2850-7200]	p<10 ^-6c^
*PCT (ng/ml)*	0.33 [0.07-1.68]	0.05 [0.03-0.1]	p<10 ^-6c^
*CRP (mg/L)*	144 [26.5-187]	6 [2-46]	p<10 ^-6c^

NLR: neutrophil-to-lymphocyte ratio; PLR: platelet-to-lymphocyte ratio; CRP: C-reactive protein; PCT: procalcitonin; ANC: absolute neutrophil count.

^*^ The significance levels were indicated as follows: a: p<0.05; b: p<0.01; c: p<0.001.

Similarly, there was a significant difference between groups with inflammatory diseases, venous thromboembolic diseases, and neoplasms ( Table 3).

**Table 3.** Kruskal-Wallis test results for comparing non-infectious disease groups ^**^.

Parameter studied	Statistic "H"	Degrees of Freedom	p-Value
NLR	10.56	2	0.005 ^c^
PLR	3	2	0.389
ANC	4.5	2	0.10 ^6^
PCT	16.6	2	<10 ^6c^
CRP	14.35	2	0.001 ^c^

NLR: neutrophil-to-lymphocyte ratio; PLR: platelet-to-lymphocyte ratio; CRP: C-reactive protein; PCT: procalcitonin; ANC: absolute neutrophil count.

^**^ The compared non-infectious disease groups were as follows: Group 1: Inflammatory Diseases; Group 2: Venous Thromboembolic Diseases; Group 3: Neoplasms.

A significant positive correlation was found between NLR and PLR and inflammation markers (CRP and PCT) (see Table 4).

**Table 4.** Pearson correlations between CRP, PCT, NLR, and PLR.

Parameter	CRP	PCT	NLR	PLR
*PLR*				
*r*	0.33	0.116	0.370	1
*p*	<10 ^−6^	0.171	<10 ^−6^	
*CRP*				
*r*	1	0.33	0.513	0.33
*p*		<10 ^−6^	<10 ^−6^	<10 ^−6^
*PCT*				
*r*	0.33	1	0.245	0.116
*p*	<10 ^−6^		0.003	0.171
*NLR*				
*r*	0.513	0.245	1	0.370
*p*	<10 ^−6^	0.003		<10 ^−6^

NLR: neutrophil-to-lymphocyte ratio; PLR: platelet-to-lymphocyte ratio; CRP: C-reactive protein; PCT: procalcitonin.

Note: Probability values (p) indicate the degree of significance of the correlation between parameters. p-values are associated with each correlation coefficient (r). A p-value less than 0.05 is considered statistically significant. The correlation coefficient (r) measures the strength and direction of the linear relationship between parameters. An r value close to 1 indicates a strong positive correlation, while an r value close to -1 indicates a strong negative correlation. An r value close to 0 suggests a weak or non-existent correlation between the parameters.

The analysis of ROC curves showed that the NLR had a sensitivity of 65.38% and a specificity of 76.47% in predicting bacterial infections in patients with clinical symptoms, with an optimal threshold value of 4.3 and an area under the curve (AUC) of 0.72 (95% CI, [0.65-0.8]. The PLR also showed prediction performance with a sensitivity of 55.3%, a specificity of 70.5%, an optimal threshold value of 183, and an AUC of 0.6 (95% CI, [0.52-0.68]).

The diagnostic performance evaluation of the four markers, namely CRP, PCT, NLR, and PLR, was conducted to predict bacterial infections in patients with clinical symptoms. CRP had the highest sensitivity and specificity with 69.23% and 88.35% respectively. The results are summarized in Table 5.

**Table 5.** Comparison of Diagnostic Performances between CRP, PCT, NLR, and PLR for Predicting Bacterial Infections.

Marker	Sensitivity	Specificity	Threshold value	Area under the Curve (AUC)
*CRP*	69.23%	88.35%	93	0.82 [0.75-0.9]
*PCT*	57.45%	83.16%	0.16	0.72 [0.62-0.82]
*NLR*	65.38%	76.46%	4.3	0.72 [0.65-0.8].
*PLR*	55.3%	70.5%	183	0,6 [0.52-0.68]

NLR: neutrophil-to-lymphocyte ratio; PLR: platelet-to-lymphocyte ratio; CRP: C-reactive protein; PCT: procalcitonin.

**Figure 1.**
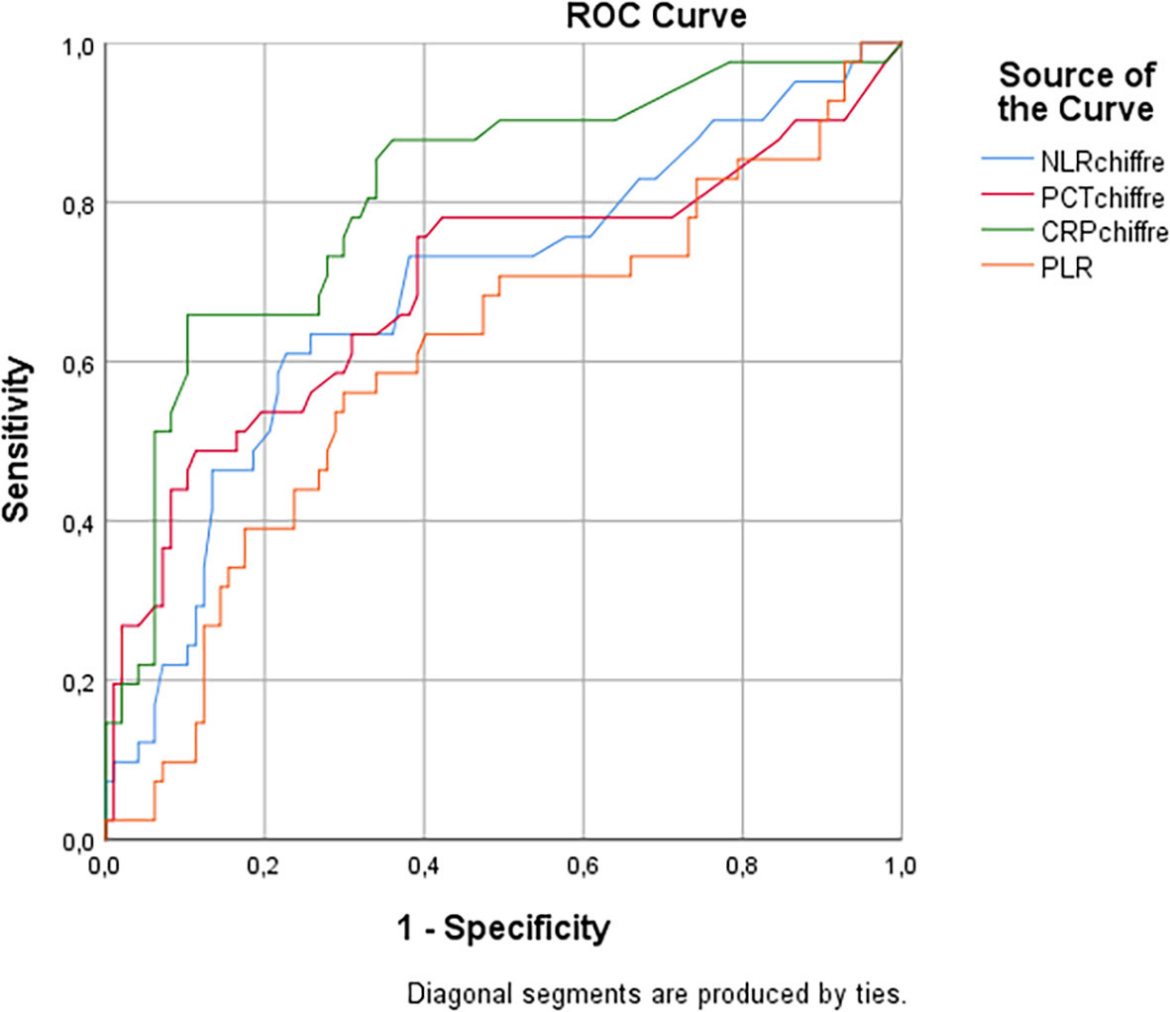
Comparison of Areas under the ROC curve for prediction of bacterial infections by CRP, PCT, NLR, and PLR markers.

The area under the ROC curve for NLR was 0.72 (95% CI, [0.65-0.8]), indicating a good diagnostic performance. Similarly, PCT had an AUC of 0.72 (95% CI, [0.62-0.82]), while PLR demonstrated an AUC of 0.6 (95% CI, [0.52-0.68]). Finally, CRP exhibited the highest AUC at 0.82 (95% CI, [0.75-0.9]) (see Figure 1).

## Discussion

Bacterial infections are a significant public health problem due to their severity and associated complications. Healthcare professionals are turning more frequently to markers of inflammation to quickly assess these infections and guide diagnostic strategies, regardless of the initial symptoms. Bacterial infections are often accompanied by a systemic inflammatory response, leading to changes in the levels of certain blood cells, including neutrophils, lymphocytes, and platelets. The increase in NLR and PLR during bacterial infections is mainly explained by the body’s inflammatory response to the infection. Neutrophils are the major immune cells involved in the defense against bacterial infections. When a bacterial infection occurs, the number of neutrophils in the blood increases rapidly in response to the bacterial invasion. Lymphocytes, on the other hand, are immune cells that play an essential role in regulating the immune response. During a bacterial infection, the number of lymphocytes may temporarily fall as some of them are mobilized to the site of infection to help fight the bacteria. The NLR therefore increases as a result of the increase in neutrophils and the temporary decrease in lymphocytes. Similarly, the PLR ratio increases because platelets, although initially designed for blood coagulation, can also be mobilized in response to inflammation. This increase in platelets can be observed during bacterial infections as a result of the body’s general inflammatory response. ^4^

Our study analyzed a group of 164 participants aged 19 to 92 years, with a male-to-female ratio of 0.76. The study population was divided into two groups: a group with confirmed bacterial infections and a group with other diagnoses. The primary objective of this study was to describe the diagnostic utility of NLR and PLR ratios in bacterial infections compared to other non-infectious causes of inflammation.

Analyses revealed that patients with bacterial infections had significantly higher levels of NLR (9.7), PLR (218.14), PCT (3.3 ng/mL), CRP (138.14mg/L), and ANC (9015.66 WBCs/μL) compared to those with non-infectious diseases. In addition, significant positive correlations were observed between NLR, PLR, and markers of inflammation.

ROC curves confirmed that NLR and PLR were good diagnostic indicators of bacterial infections, with AUC values of 0.72 and 0.6, respectively. These results highlight the importance of these ratios in the management of patients with bacterial infections.

Clinical studies, such as the one conducted by Zahorec and al. on patients with confirmed bacterial infections, have demonstrated a significant increase in the NLR compared to reference values, suggesting its utility as a reliable indicator. ^9^ Similarly, Wang and al. have investigated the potential of the PLR as an indicator of systemic inflammation, with a specific focus on sepsis cases. Their findings suggested that PLR could indeed serve as a valuable prognostic indicator for sepsis and a significant association between elevated PLR values and unfavorable outcomes in sepsis patients was demonstrated. ^10^

The use of NLR and PLR markers is crucial for a rapid identification of bacterial infections, particularly when initial clinical manifestations are inconspicuous. These markers offer an interesting insight into the patient’s immune and inflammatory status at the beginning of their admission, allowing earlier and more accurate management. Identifying substantial deviations from reference values enables healthcare professionals to promptly suspect an underlying bacterial infection, even with mild symptoms. This proactive approach helps managing ulterior investigations and monitoring the effectiveness of treatments. In consequence, clinicians could predict severe complications and enhance clinical outcomes. ^11^^,^^12^

Our results revealed significant variations in NLR based on gender. Indeed, men generally had slightly higher NLR values than women, with means of 7.2 in men and 4.7 in women (p < 10 ^-6^), consistent with trends observed in other studies, including the one conducted by Fors and al. In this large-scale study, NLR values were on average higher in men (median of 2.69) than in women (median of 2.35) with a p value < 0.001. ^13^ Additionally, our identification of age-related differences, with older individuals exhibiting elevated NLR levels (mean of 8.8) compared to their younger counterparts, is in agreement with the broader literature on age-related changes in inflammatory markers. ^14^

This observation of NLR variations by gender underscores the importance of considering this difference when interpreting results and evaluating the diagnosis of bacterial infections.

Furthermore, the results of our study indicate that these markers have an important diagnostic utility in bacterial infections. Indeed, NLR and PLR values, along with levels of PCT, and CRP, were significantly higher in patients with bacterial infections compared to those with other pathologies, which is in line with the literature data. ^15^^–^^18^

To illustrate, in our study, the means values were as follows: PCT, 3.2 ng/mL; CRP, 152 mg/L; NLR, 9.7; PLR, 218. These results are consistent with several other recent studies on bacterial infections. In another study by Li and colleagues, the median PCT in patients with bacterial infections was similar, with a value of 0.67 ng/mL. ^19^ Additionally, Ding and al also found comparable results for NLR, with a median of 8.7 in patients with bacterial infections. ^2^ Similarly, Yang and colleagues reported a mean PLR of 169.5 in patients with bacterial infections, which corroborates our findings. ^20^

However, it is important to note that in the non-infectious pathology group, we observed significantly lower levels, with mean values of PCT, NLR, and PLR of 0.21 ng/mL, 3.9, and 174, respectively.

In the context of our study, we also evaluated the diagnostic performance of NLR, PLR, and CRP in predicting bacterial infections using the Receiver Operating Characteristic (ROC) curve areas (AUC). Our results showed that NLR had an AUC of 0.72, while PLR had an AUC of 0.6. Although these values indicate a reasonable ability of these markers to differentiate bacterial infections from other pathologies, CRP showed the highest AUC of 0.82, highlighting its superior effectiveness in predicting bacterial infections.

These findings are in line with previous works. For example, Cai and colleagues’ study in 2017 which evaluated NLR in predicting bacterial infections in patients with decompensated cirrhosis, confirmed a significant AUC value of approximately 0.824. In this retrospective study involving 2066 decompensated cirrhotic patients, the incidence of hospital-acquired (HA) bacterial infections was approximately 35.87% in the training cohort and 31.05% in the validation cohort. Multivariate analysis identified total bilirubin, albumin, white blood cell count, and NLR as independent predictors of HA bacterial infections. These results reinforce the importance of NLR as a valuable and noninvasive marker for early detection and prediction of hospital-acquired bacterial infections in individuals with decompensated cirrhosis. ^21^ Similarly, Thill and colleagues’ study (2019) examined the diagnostic value of NLR in the context of fever and inflammatory syndromes in internal medicine, reinforcing our findings regarding NLR. ^4^ In this prospective study of 184 hospitalized patients in internal medicine, the NLR was assessed as a diagnostic marker to differentiate bacterial infections from other causes of fever and inflammation. The study revealed that NLR, with an optimal threshold of seven, showed significance in the presence of 82 bacterial infection cases, and its Area Under the ROC Curve (AUC) was 0.64, comparable to CRP and PCT in this clinical context.

It is important to note that CRP has consistently demonstrated superior diagnostic performance in the context of bacterial infections. In this regard, Legrain and al. (2017) also contributed to the understanding of CRP’s effectiveness as a distinct indicator of bacterial infections. ^22^

In contrast, another study conducted by Shanshan Ding et al. yielded contradictory results to ours. This retrospective clinical study aimed to differentiate infectious fever from tumor fever (TF) and assess outcomes in non-neutropenic lung cancer patients (NNLCPs) by evaluating PCT, CRP, and NLR as markers of inflammation. ^2^ The study included 588 febrile NNLCPs, of which 311 had bacterial infections, and 277 had TF. Inflammatory markers (PCT, CRP, white blood cells, neutrophils, and NLR) were significantly higher in bacterial infection cases (p < 0.0001). PCT demonstrated the highest predictive value for bacterial infections (AUC = 0.874), followed by CRP (AUC = 0.855) and NLR (AUC = 0.792) (p < 0.0001). In conclusion, PCT outperformed CRP and NLR in diagnosing bacterial infections in febrile patients and proved useful in assessing clinical outcomes and cancer progression in NNLCPs.

In summary, our results on the AUCs for NLR, PLR, and CRP align with the conclusions of previous studies, confirming the variable performance of these markers in predicting bacterial infections. CRP remains the most performant marker, while NLR and PLR retain reasonable diagnostic utility. This understanding can guide clinical decision-making for the precise diagnosis of bacterial infections, taking into account the context and individual patient characteristics.

However, it is important to note that this study has certain limitations. The relatively small sample size may restrict the generalizability of the results to larger populations. Additionally, it is essential to consider the variability in results based on the nature of bacterial infections and the clinical characteristics of patients.

## Conclusion

In conclusion, this study has demonstrated that NLR and PLR could have a significant diagnostic role in bacterial infections. These markers could contribute to improving the accuracy of diagnosis, assessing the severity of infection, and guiding therapeutic decisions. However, further research and larger studies are needed to confirm and explore these promising results.

### Ethics and consent

Ethical approval was obtained from the ethics committee of Sahloul University Hospital of Sousse on May 11, 2022, under the number **HS 22-2022**, this approval confirms that there are no ethical issues concerning the work. Written and verbal consent were obtained from all participants before their involvement in the study, ensuring that their rights, privacy, and confidentiality were rigorously upheld throughout the research process.

## Data Availability

Dryad: Data from: The interest of inflammatory biomarkers in the diagnostic approach, https://doi.org/10.5061/dryad.n02v6wx3d. ^23^ This project contains following dataset:
1.
Data_Inflammatory_Biomarkers_1.sav 2. README Data are available under the terms of the Creative Commons Zero “No rights reserved” data waiver (CC0 1.0 Public domain dedication).
