# Supplementary material for: Neutrophil-to-lymphocyte and platelet-to-lymphocyte ratios in bacterial infections: contributions to diagnostic strategies in a tertiary care hospital in Tunisia
Source: F1000Res. 2024 Sep 25;13:978. Originally published 2024 Aug 29. [Version 2] doi: 10.12688/f1000research.146952.2 (PMC11408912; doi:10.12688/f1000research.146952.2)
Supplement: Peer review history

# Reviewer report: Reviewer response for version 2

**DOI:** 10.5256/f1000research.172231.r326824

**Published:** 2024-09-25

**Authors:** Saoussen Chouchene

**Affiliations:** 1 Fattouma Bourguiba University Hospital,, Monstir,, Tunisia

Thank you for the update regarding the revised version of the manuscript titled *"Neutrophil-to-lymphocyte and platelet-to-lymphocyte ratios in bacterial infections: contributions to diagnostic strategies in a tertiary care hospital in Tunisia"*. I have reviewed the changes made by the authors in response to the peer review feedback, and I find the modifications satisfactory. As such, I confirm my approval of the revised article.

Is the work clearly and accurately presented and does it cite the current literature?

Yes

If applicable, is the statistical analysis and its interpretation appropriate?

Yes

Are all the source data underlying the results available to ensure full reproducibility?

Yes

Is the study design appropriate and is the work technically sound?

Yes

Are the conclusions drawn adequately supported by the results?

Yes

Are sufficient details of methods and analysis provided to allow replication by others?

Yes

Reviewer Expertise:

Hematology and immunogenetics

I confirm that I have read this submission and believe that I have an appropriate level of expertise to confirm that it is of an acceptable scientific standard.

# Reviewer report: Reviewer response for version 1

**DOI:** 10.5256/f1000research.161084.r318818

**Published:** 2024-09-24

**Authors:** Wissal Ben Yahia

**Affiliations:** 1 University of Sousse Faculty of Medicine of Sousse (Ringgold ID: 280226), Sousse, Tunisia; 2 Farhat Hached University Hospital of Sousse (Ringgold ID: 280368), Internal Medicine department, Sousse, Sousse, Tunisia

I congratulate all authors for their strong choice of subject and the quality of the manuscript. 

 I have just some remarks to make better your work :

 1. I suggest to add "Bacteria" or "bacterial infection" as a keyword.

 2. There are 2 abbreviations that were explained for the first time in the discussion but were present in the abstract (AUC) and in results for (ROC) so put the explanations on their first appearance.

 3. You should add in excluded criteria the exclusion of viral infections and parasitic.

 4. exclude the number of the population from the methodology because its a result " a prospective comparative study was conducted within the internal medicine department on patients admitted between January 1st and July 31st, 2023."  

 These remarks will just improve your work and do not decrease in any way its rigour and quality.

Is the work clearly and accurately presented and does it cite the current literature?

Yes

If applicable, is the statistical analysis and its interpretation appropriate?

Yes

Are all the source data underlying the results available to ensure full reproducibility?

Yes

Is the study design appropriate and is the work technically sound?

Yes

Are the conclusions drawn adequately supported by the results?

Yes

Are sufficient details of methods and analysis provided to allow replication by others?

Yes

Reviewer Expertise:

Systemic diseases as vasculitis and connectivitis, treatment and etiologic exploration of thrombosis, and exploration of general signs as fever, weight loss, nodes.. medical education, Simulation, Statistical analyses

I confirm that I have read this submission and believe that I have an appropriate level of expertise to confirm that it is of an acceptable scientific standard.

# Reviewer report: Reviewer response for version 1

**DOI:** 10.5256/f1000research.161084.r318817

**Published:** 2024-09-17

**Authors:** Aya Chakroun

**Affiliations:** 1 La Rabta University Hospital, Tunis, Tunisia

I congratulate you on the choice of subject and the quality of the manuscript. However, there are a few improvements that should be made:

- In the ”methods” section:

-Please remove ‘’ To address the research question’’

 - Please remove the expression “The study included a total of 164 patients “ .

 - Please add to the section “Laboratory values and clinical assessment”, an indication of good control of the analytical process through the daily passage of internal quality controls.

- In the “results” section:

-please add “footnotes” to the tables, explaining abbreviations and add confidence intervals of r.

 I congratulate you for your scientific rigour and the quality of the discussion of the relevant scientific literature.

Is the work clearly and accurately presented and does it cite the current literature?

Yes

If applicable, is the statistical analysis and its interpretation appropriate?

Yes

Are all the source data underlying the results available to ensure full reproducibility?

No source data required

Is the study design appropriate and is the work technically sound?

Yes

Are the conclusions drawn adequately supported by the results?

Yes

Are sufficient details of methods and analysis provided to allow replication by others?

Yes

Reviewer Expertise:

areas of expertise: benign and malignant hematologyHematology analyzer technologiesmedical educationStatistical analyses and biomedical research

I confirm that I have read this submission and believe that I have an appropriate level of expertise to confirm that it is of an acceptable scientific standard.

# Response

**Published:** 2024-09-17

**Authors:** Salma Riahi

We greatly appreciate your time and effort in reviewing and approving our article. Your insightful suggestions and feedback are incredibly valuable to us, and we are fully dedicated to implementing the necessary changes to enhance the quality of the writing in accordance with your recommendations. Your input is instrumental in helping us refine and improve our work, and we are truly grateful for your support.

# Reviewer report: Reviewer response for version 1

**DOI:** 10.5256/f1000research.161084.r318819

**Published:** 2024-09-13

**Authors:** Saoussen Chouchene

**Affiliations:** 1 Fattouma Bourguiba University Hospital,, Monstir,, Tunisia

This study examines the utility of two hematological markers, the neutrophil-to-lymphocyte ratio (NLR) and platelet-to-lymphocyte ratio (PLR), in diagnosing bacterial infections. Below is a detailed summary and suggestions for improvement.

**Article Summary**

**Introduction:**

 Bacterial infections are a significant global health challenge, contributing to mortality through antibiotic resistance and complications such as septicemia. While traditional diagnostic methods like pathogen isolation are reliable, they may be impractical or slow. Biomarkers like C-reactive protein (CRP), procalcitonin (PCT), NLR, and PLR offer a simpler, cost-effective means of identifying infections early. NLR and PLR are particularly attractive due to their accessibility.

**Methods:**

 This prospective study included 164 adult patients admitted to the internal medicine department from January 1st to July 31st, 2023, all of whom had not received prior antibiotics or immunosuppressive therapy. Lab values, including neutrophil, platelet, lymphocyte counts, CRP, and PCT levels, were recorded. NLR and PLR ratios were calculated and compared between patients with bacterial infections and those with non-infectious inflammatory conditions. Diagnostic performance was evaluated using ROC curves through SPSS and MedCalc.

**Results:**

 Patients with bacterial infections had elevated NLR and PLR. Differences were observed across age and gender, with higher NLR levels in men and older patients. ROC analysis demonstrated moderate diagnostic accuracy for NLR and PLR, with CRP showing the highest accuracy in this population.

**Discussion:**

 The study confirms that NLR and PLR are useful diagnostic tools for bacterial infections, especially when combined with CRP and PCT. These markers are particularly valuable in settings with limited access to advanced diagnostic technologies. However, CRP remains the most reliable marker. Additionally, patient characteristics like age and gender should be considered when interpreting these values.

**Conclusion:**

 NLR and PLR hold potential as diagnostic tools for bacterial infections, but further large-scale studies are required to validate these findings. CRP continues to be the most effective biomarker.

**Suggestions for Improvement**

**Introduction:**

- **Strengths:** The rationale for studying NLR and PLR alongside traditional markers is clear.
- **Suggestions:** Further elaborate on the limitations of existing diagnostic methods and explain why NLR and PLR may be particularly useful in certain clinical contexts.

**Methods:**

- **Strengths:** Clear methodology and well-defined inclusion/exclusion criteria.
- **Suggestions:**
  - Provide more details on how bacterial infections were confirmed.
  - The small sample size limits generalizability—address this limitation.
  - Consider the impact of confounding factors such as chronic inflammatory diseases and immune disorders that may affect NLR and PLR.

**Results:**

- **Strengths:** Clearly presented results with appropriate statistical analysis.
- **Suggestions:** Comparing ROC curve findings with similar studies would strengthen the discussion.

**Discussion:**

- **Strengths:** Comprehensive discussion linking findings with existing literature.
- **Suggestions:**
  - Critically evaluate the limitations of NLR and PLR, including potential situations where these markers may be less effective.
  - Explore the clinical relevance of these findings in resource-constrained settings.
  - Offer practical advice for interpreting NLR and PLR in light of patient age and gender differences.

**Limitations:**

- Expand the limitations section, addressing possible alternative explanations for elevated NLR and PLR and the potential for other inflammatory conditions to mimic these findings.

**Ethical Considerations:**

- Adequately addressed; no further comments needed.

**Conclusion**

 This study provides valuable insights into the diagnostic utility of NLR and PLR in bacterial infections. Expanding on confounding factors, refining the clinical implications, and addressing study limitations will improve its scientific rigor. This research significantly contributes to understanding the diagnostic potential of hematological markers, though further studies are encouraged.

**Acceptance Statement:**

 After thorough analysis, I recommend this article for publication, with minor revisions. Its findings are compelling and make a valuable contribution to the field. Expanding the discussion and addressing confounding factors will enhance its overall impact.

Is the work clearly and accurately presented and does it cite the current literature?

Yes

If applicable, is the statistical analysis and its interpretation appropriate?

Yes

Are all the source data underlying the results available to ensure full reproducibility?

Yes

Is the study design appropriate and is the work technically sound?

Yes

Are the conclusions drawn adequately supported by the results?

Yes

Are sufficient details of methods and analysis provided to allow replication by others?

Yes

Reviewer Expertise:

Hematology and immunogenetics

I confirm that I have read this submission and believe that I have an appropriate level of expertise to confirm that it is of an acceptable scientific standard.

# Response

**Published:** 2024-09-17

**Authors:** Salma Riahi

We express our sincere gratitude for your comprehensive review and approval of our article. Your discerning recommendations have provided us with a clear roadmap for enhancement, and we deeply value the commitment and diligence you have shown in furnishing such meticulous feedback. We are committed to effecting all requisite revisions to elevate the caliber of the composition and enhance the lucidity of our discourse. Your guidance has been instrumental and we are eager to integrate these modifications in accordance with your suggestions.
